# NUCKS1, a novel Tat coactivator, plays a crucial role in HIV-1 replication by increasing Tat-mediated viral transcription on the HIV-1 LTR promoter

**DOI:** 10.1186/s12977-014-0067-y

**Published:** 2014-08-13

**Authors:** Hye-Young Kim, Byeong-Sun Choi, Sung Soon Kim, Tae-Young Roh, Jihwan Park, Cheol-Hee Yoon

**Affiliations:** Division of AIDS, Korean National Institute of Health, Chungbuk, Republic of Korea; Department of Life Sciences, Pohang University of Science and Technology (POSTECH), Pohang, 790-784 Republic of Korea

**Keywords:** HIV-1 Tat, NUCKS1, HIV-1 transcription, LTR, TAR-RNA

## Abstract

**Background:**

Human immunodeficiency virus-1 (HIV-1) Tat protein plays an essential role in HIV gene transcription from the HIV-1 long terminal repeat (LTR) and replication. Transcriptional activity of Tat is modulated by several host factors, but the mechanism responsible for Tat regulation by host factors is not understood fully.

**Results:**

Using a yeast two-hybrid screening system, we identified Nuclear ubiquitous casein and cyclin-dependent kinase substrate 1 (NUCKS1) as a novel Tat-interacting partner. Here, we report its function as a positive regulator of Tat. In a coimmunoprecipitation assay, HIV-1 Tat interacted sufficiently with both endogenous and ectopically expressed NUCKS1. In a reporter assay, ectopic expression of NUCKS1 significantly increased Tat-mediated transcription of the HIV-1 LTR, whereas knockdown of NUCKS1 by small interfering RNA diminished Tat-mediated transcription of the HIV-1 LTR. We also investigated which mechanism contributes to NUCKS1-mediated Tat activation. In a chromatin immunoprecipitation assay (ChIP), knockdown of NUCKS1 interrupted the accumulation of Tat in the transactivation-responsive (TAR) region on the LTR, which then led to suppression of viral replication. However, NUCKS1 expression did not increase Tat nuclear localization and interaction with Cyclin T1. Interestingly, the NUCKS1 expression level was lower in latently HIV-1-infected cells than in uninfected parent cells. Besides, expression level of NUCKS1 was markedly induced, which then facilitated HIV-1 reactivation in latently infected cells.

**Conclusion:**

Taken together, our data demonstrate clearly that NUCKS1 is a novel Tat coactivator that is required for Tat-mediated HIV-1 transcription and replication, and that it may contribute to HIV-1 reactivation in latently HIV-1 infected cells.

**Electronic supplementary material:**

The online version of this article (doi:10.1186/s12977-014-0067-y) contains supplementary material, which is available to authorized users.

## Background

Human immunodeficiency virus type-1 (HIV-1) encodes a transcriptional activator (transactivator) protein, Tat, which is essential for efficient transcription of the integrated provirus following HIV-1 replication and for disease progression [1,2]. Tat binds cooperatively to transactivation-responsive (TAR) RNA, which is found at the 5’ end of all HIV-1 viral RNAs with cellular factors, followed by transcriptional elongation [3]. Several host cellular factors are essential for the transcriptional activity of Tat. Positive transcription elongation factor b (P-TEFb) composed of Cyclin T1 (CycT1) and CDK9 is one of the most important factors promoting HIV-1 transcription. P-TEFb is connected to other transcription elongation activators/co-activators including ELL2 and ENL/AF9 by scaffold protein AFF4/AFF1 to form a larger super elongation complex (SEC). Tat selectively recruits SEC to HIV-1 LTR to stimulate viral transcription [4–7]. Subsequently, both elongation activators (P-TEFb and ELL2) cooperatively activate processive HIV-1 transcription through phosphorylation of the C-terminal domain of RNA polymerase II (Pol II) and by preventing of its backtracking [8,9]. Although it has also been reported that Tat activity is modulated by Tat-interacting proteins such as Tip60, HT2A, CA150, TFIID, Tat-SF1, Tip30, p300, CREB-binding protein, PC4, and Tip110, which regulate Tat activity by regulating its interactions with TAR RNA and/or other host factors [10–20], the precise mechanisms underlying Tat regulation by these factors remain unclear. A number of cellular transcription factors such as SP1, nuclear factor κB (NF-κB), TBP, nuclear factor of activated T cells (NFAT), USF, and nuclear receptor chicken ovalbumin upstream promoter transcription factor (COUP-TF), also regulate the HIV-1 LTR promoter activity through target elements located upstream of TAR RNA within the LTR promoter [21–25].

HIV-1 gene expression is regulated by epigenetic control by histone-modifying enzymes (HDAC, PCAF, GCN5, SUV39H1, etc.), DNA methylation on the CpG islands in the LTR, and nucleosome remodeling by SWI/SNF of HIV-1 integrase [26–28]. Thus, it is believed that Tat–host cellular factor interactions, transcription, and epigenetic factors form a complex regulatory network for the regulation of HIV-1 gene expression in a diverse range of host cells and in response to a variety of extracellular stimuli. Therefore, identification of key factors for Tat-mediated HIV-1 gene expression may be required for understanding HIV replication and for exploring potential targets of antiviral drugs [29,30].

Nuclear ubiquitous casein and cyclin-dependent kinase substrate 1 (NUCKS1) is a nuclear DNA-binding protein that is highly phosphorylated by casein kinase II (CK2), cyclin-dependent kinases 1 (Cdk1), and DNA-activated kinase [31,32]. NUCKS1 is expressed ubiquitously in all mammalian tissues, and its apparent molecular mass is ~28.4 kDa including posttranslational modifications. However, its molecular mass appears to be 50 kDa in sodium dodecyl sulfate-polyacrylamide gel electrophoresis (SDS-PAGE) [32]. NUCKS1 is synthesized in the M/G1 phase [33] and is highly expressed in several human cancers including ovarian, lung, bone marrow, brain, and breast cancer [34–36]. Even though NUCKS1 seems to be important to the cell cycle and cancer progression, its exact role has not been clarified.

We used the yeast two-hybrid screening system to explore the Tat regulatory mechanism in relation to the host factors. Here, we report that NUCKS1 is a novel Tat-interacting partner that induces Tat-mediated viral transcription by accumulating Tat on TAR RNA regions of the LTR promoter, which may then contribute to the positive regulation of viral replication in HIV-1 latently infected cells during reactivation.

## Results

### NUCKS1 and Tat interact

**Figure 1:**
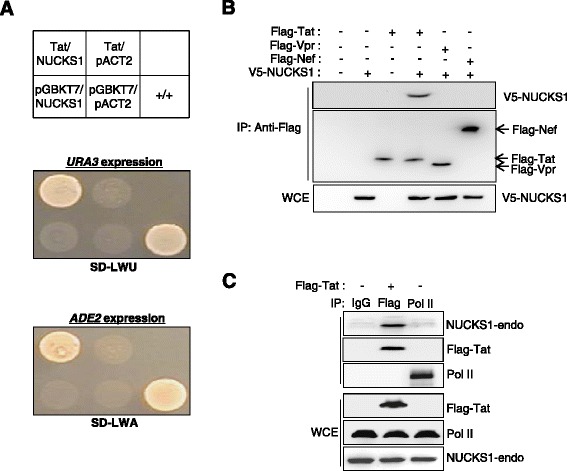
**Interaction between HIV-1 Tat and NUCKS1. (A)** Tat-expressing plasmid cloned into pGBKT7 and NUCKS1 selected from human thymus cDNA library cloned into pACT2 (Clontech) was transformed into the yeast strain PBN204. The transformants were grown on an SD plate lacking adenosine and uracil for 48 h. Murine p53 and SV40 large T antigens were used as a positive control. Two panels show yeast colonies showing the interaction of Tat and NUCKS1. **(B)** Ectopically expressed Tat interacts with ectopically expressed NUCKS1 in HEK293 cells. Two micrograms of the Flag–Tat, Flag-Vpr and Flag-Nef expression vector were cotransfected with the V5–NUCKS1 expression vector into HEK293 cells cultured in 100 mm plates. Forty-eight hours after transfection, cell lysates were immunoprecipitated with an anti-Flag monoclonal antibody (M2). Immunoprecipitates were analyzed by Western blotting using an HRP-conjugated anti-V5 monoclonal antibody. The Flag- or V5-tagged pcDNA3 plasmid was added to equalize the total amounts of DNA. **(C)** Coimmunoprecipitation assay between endogenous NUCKS1 and ectopically expressed Tat protein. The lysate from Tat-expressing HEK293T cells was immunoprecipitated with anti-Flag and -Pol II antibodies, and the interaction was assessed with Western blotting using anti-NUCKS1 or anti-Flag antibody.

To identify the binding partners of HIV-1 Tat, we used a yeast two-hybrid screening with full-length HIV-1 Tat (amino acids 1–86) as the bait together with the human thymus cDNA library as the prey. From 44 positive clones resulting from nutritional selection, one clone was found to contain a cDNA of 732 bp (GenBank accession number NM_022731) encompassing the full-length open-reading frame of the NUCKS1 protein (Figure 1A). To determine whether Tat interacts with cellular NUCKS1, Flag-tagged HIV-1 proteins (Flag–Tat, -Vpr and -Nef) and V5-tagged NUCKS1 (V5–NUCKS1) expression plasmids were introduced into HEK293 cells individually (with each empty vector) or in combination and then analyzed by coimmunoprecipitation and a Western blot assay. Tat coimmunoprecipitated with NUCKS1 in cells expressing both Flag-Tat and V5-NUCKS1, but other HIV proteins (Vpr and Nef) did not (Figure 1B). Next, we tried to confirm the endogenous interaction between Tat and NUCKS1. As shown in Figure 1C, endogenous NUCKS1 was immuno-precipitated by the anti-Flag antibody in Flag–Tat-expressing HEK293 cells, but not by the RNA polymerase II antibody (Figure 1C). These results suggest that Tat interacts directly with both ectopically expressed and endogenous NUCKS1.

### NUCKS1 facilitates Tat-mediated transcriptional activation on the HIV-LTR

**Figure 2:**
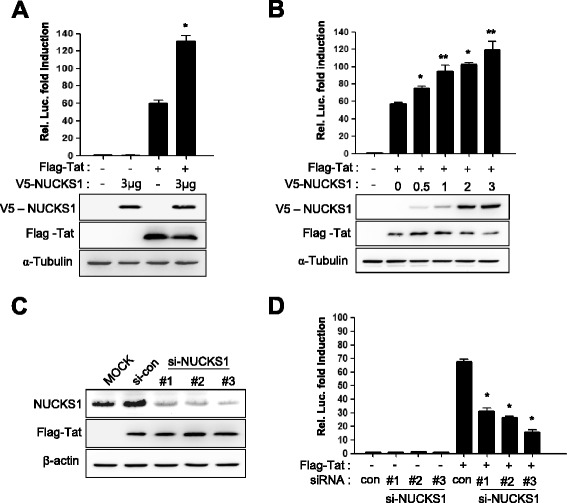
**NUCKS1-mediated Tat activation on HIV-1 LTR. (A)** One hundred nanograms of the Flag–Tat and/or 3 μg of the NUCKS1 expression plasmid was transfected with 200 ng of the HIV-1 LTR-driven luciferase reporter (pGL3–LTR–Luc) and 20 ng of pCMV–LacZ as a transfection control into HeLa cells. At 24 h after transfection, luciferase activity was measured and normalized to β-galactosidase activity. The data are expressed as mean ± SD (n = 3). *, *P* < 0.01 as compared with the Flag-Tat transfected only. Expression levels of NUCKS1 and Tat were assessed with Western blotting. **(B)** Luciferase activity was assessed in HeLa cells 24 h after cotransfection of pGL3–LTR–Luc (200 ng), Flag–Tat (100 ng), and increasing amounts of V5–NUCKS1 (0, 0.5, 1, 2, and 3 μg), together with 20 ng of pCMV–LacZ as a transfection control. The data are expressed as mean ± SD (n = 3). *, *P <* 0.01 and **, *P* < 0.05, as compared with the Flag-Tat transfected only **(C)** Knockdown of NUCKS1 was performed with three siRNAs against NUCKS1 mRNA. HeLa cells were transfected with control siRNA or three NUCKS1 siRNAs. At 48 h after transfection, knockdown of NUCKS1 by siRNA was assessed by Western blotting using anti-NUCKS1, anti-Flag and anti-β-actin antibodies as a loading control. **(D)** NUCKS1-engaed Tat activity was assessed by the luciferase assay combined with the knockdown experiment. HeLa cells were transfected with control siRNA or three NUCKS1 siRNAs. Twenty-four hours after transfection, cells were transfected further with pGL3–LTR–Luc, pCMV–*LacZ*, and Flag–Tat. The luciferase assay was conducted as described in A. The data was expressed as mean ± SD (n = 3). *, P < 0.01 as compared with the cells transfected with control siRNA.

To determine whether the NUCKS1 has a functional role in Tat-induced transcriptional activation on the HIV-1 LTR, HIV-1 LTR-driven luciferase and the NUCKS1 expression plasmid were introduced into HeLa cells in the presence or absence of the Tat expression plasmid. Even though Tat markedly increased the transcription of LTR, expression of NUCKS1 increased this activity further by more than 2.3-fold (Figure 2A). NUCKS1 alone had no effect on the HIV LTR in the absence of Tat (Figure 2A). Expression of increasing amounts of NUCKS1 increased the Tat-mediated transcriptional activity on the LTR in a dose-dependent manner without changing the amount of Tat expressed (Figure 2B).

To examine the effect of the loss of function of NUCKS1 in Tat-mediated transcriptional activation of the HIV-1 LTR, knockdown of NUCKS1 was performed with three siRNAs against NUCKS1, which effectively decreased the expression level of NUCKS1 (Figure 2C). Tat-mediated LTR activation was decreased significantly by NUCKS1 knockdown with these siRNAs, whereas NUCKS1 knockdown had no effect in the absence of Tat, which was consistent with that showing no effect of NUCKS1 expression (Figure 2D). These data demonstrate that NUCKS1 plays a crucial role in the Tat-mediated transcription on the HIV-1 LTR and suggest that Tat–NUCKS1 interaction may be required for this role.

### NUCKS1-mediated Tat activation is not cause by activation of NF-κB, Tat nuclear localization and Tat–Cyc T1 interaction

**Figure 3:**
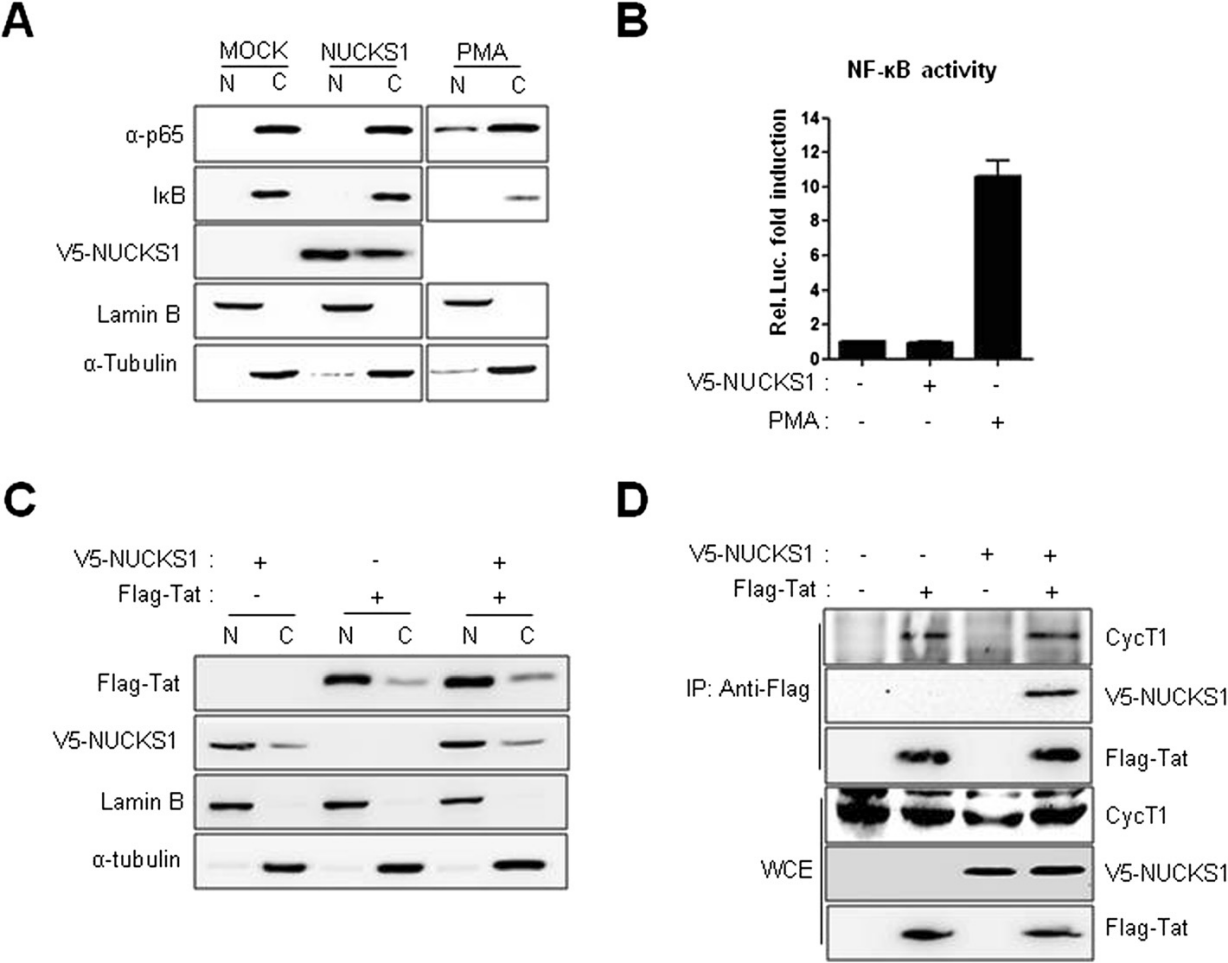
**Mechanism linked to NUCKS1-mediated Tat activation. (A)** HeLa cells were transfected with the V5–NUCKS1 expression plasmid. At 24 h after transfection, some of the cells were treated with PMA (50 ng/ml) for 15 min before harvest, and the cells were harvested. Nucleus and cytoplasm from the cells were fractionated. NF-κB activity was assessed by Western blotting using anti-p65, -Iκb, and -NUCKS1 antibodies. The nuclear and cytoplasmic fractions were evaluated using anti-lamin B and -tubulin antibodies, respectively. **(B)** NUCKS1-mediated NF-κB promoter activity was assessed by luciferase assay. HeLa cells transfected with NF-κB luciferase reporter plasmid (500 ng), V5–NUCKS1 (3 μg), and pCMV–*LacZ* (20 ng). The luciferase assay was performed 24 h after transfection. PMA (50 ng/ml) treatment was used as a positive control. The data are expressed as mean ± SD (n = 3). **(C)** HeLa cells were cotransfected with the Flag–Tat and V5–NUCKS1 expression plasmids. Twenty-four hours after transfection, the nucleus and cytoplasm were fractionated, and the subcellular localization of Tat and NUCKS1 was assessed with Western blotting using anti-Flag, anti-V5, -lamin B, and -α-tubulin antibodies, respectively. **(D)** HeLa cells were transfected with Flag–Tat, V5–NUCKS1, or both. Two days after transfection, cell lysates were immunoprecipitated with anti-Flag antibody. Cyc T1–Tat interaction was assessed with Western blotting using anti-Cyclin T1 and anti-Flag antibodies, respectively.

We next explored the mechanism by which NUCKS1 regulates Tat. Since NF-κB activation is required in the early step of HIV-1 LTR transcription, we examined whether NUCKS1 could regulate NF-κB activation. As shown in Figure 3A, neither nuclear p65 nor cytoplasmic IκB levels changed when NUCKS1 was expressed. This contrasted with the effects of phorbol myristate acetate (PMA) treatment following p65 nuclear localization and degradation of IκB (Figure 3A). As a complementary approach, we assessed the NF-κB response element (RE)-driven promoter activity in association with NUCKS1 expression and PMA treatment in HeLa cells. A promoter harboring the NF-κB RE was not responsible for NUCKS1 expression, whereas its activity was increased markedly by PMA treatment (Figure 3B). These data indicate that NF-κB is not involved in NUCKS1-mediated Tat activation.

We next attempted to determine whether NUCKS1 facilitates Tat nuclear localization because NUCKS1 is a known nuclear protein containing two nuclear-localization signals [31]. As shown in Figure 3C, both NUCKS1 and Tat localized mainly to the nucleus when expressed either alone or together in HeLa cells (Figure 3C). Cyc T1 interaction with Tat is essential for HIV-1 transcription. We next wanted to determine whether NUCKS1-mediated Tat activation is caused by enhancement of Tat–Cyc T1 interaction. We introduced the Flag–Tat and V5–NUCKS1 expression plasmids into HeLa cells, as shown in Figure 3D. Flag–Tat was then immunoprecipitated from the cell lysates. Tat bound simultaneously to NUCKS1 and Cyc T1, and the Tat–Cyc T1 interaction was not interrupted by NUCKS1 expression (Figure 3D). These data suggest that NUCKS1-mediated Tat activation may not be associated with the increase in Tat–Cyc T1 interaction.

### NUCKS1 enhances the association of Tat to the TAR sequence

**Figure 4:**
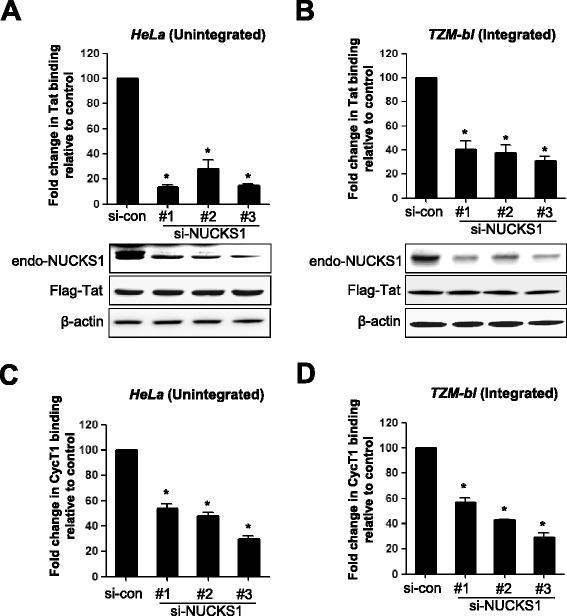
**Close association between NUCKS1 and Tat binding to TAR RNA.** HeLa cells transiently transfected with HIV-1 LTR **(A and C)** or TZM-bl cells containing an integrated HIV-1 LTR **(B and D)** were transfected with control siRNA or three individual NUCKS1 siRNAs. At 24 h after knockdown, these cells were transiently transfected with the Flag–Tat expression plasmid and cultured for an additional 24 h. Cells were cross-linked with formaldehyde, and chromatin immunoprecipitation was performed using IgG control or anti-Flag antibody **(A and B)** or anti-Cyclin T1 **(C and D)**. Quantitative real-time PCR was performed; the data were normalized to the IgG control antibody and are expressed as fold change of Tat binding activity compared with control siRNA-treated samples. The data are expressed as mean ± SD (n = 3). *, *P* < 0.01 as compared to the cells transfected with control siRNA.

To identify the possible mechanism underlying NUCKS1-mediated Tat activation of the HIV-1 LTR, we next used a ChIP assay with knockdown of NUCKS1. NUCKS1 siRNA was transfected into HeLa cells introduced with the HIV-1 LTR, and chromatin was then immunoprecipitated with Tat. A chromatin coding TAR RNA in LTR was immuno-precipitated with Tat, while other regions were not (Additional file 1: Figure S1A). The depletion of NUCKS1 by three independent siRNAs significantly reduced the level of Tat associated with the chromatin coding TAR RNA, conceivably by reducing the amount of Tat bound to TAR RNA (Figure 4A). Similar effects were observed in TZM-bl cells integrated with HIV LTR-driven β-galactosidase and luciferase reporter cassettes (Figure 4B and Additional file 1: Figure S1B). The reduction of the amount of Cyclin T1 in the TAR-RNA region, which might have been caused by the loss of Tat in LTR, was observed by NUCKS1 knockdown in both cells (Figure 4C and D). These data suggest that NUCKS1 is closely associated with the interaction between Tat and the TAR RNA region in both integrated and unintegrated LTRs during Tat-induced HIV-1 transcription.

### NUCKS1 plays a key role in viral production

**Figure 5:**
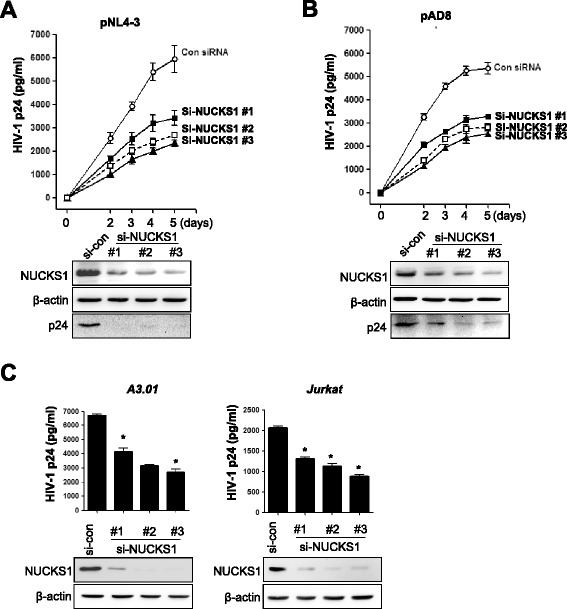
**Key role of NUCKS1 in viral production and replication. (A-B)** In the single-round replication assay, HeLa cells were transfected with control siRNA or three individual NUCKS1 siRNAs. One day later, the cells were transfected with pNL4-3 **(A)** or pAD8 **(B)** proviral DNA. Two days after siRNA transfection, the virus-containing supernatants were harvested every day for 5 days. The amount of virion in the supernatant was measured by HIV-1 p24 ELISA. **(C)** In the viral replication assay, Jurkat and A3.01 cells were transfected with control siRNA or three individual NUCKS1 siRNAs. One day later, the cells were infected with pNL4-3 virus. Five days after infection, the virus-containing supernatants were harvested, and then the amount of virions in the supernatant was measured by HIV-1 p24 ELISA. The data are expressed as mean ± SD (n = 3). *, *P* < 0.01 as compared with the cells transfected with control siRNA. The lower panels show the intracellular p24 levels.

Because HIV-1 Tat activity is essential for HIV-1 production and replication, we thought that NUCKS1-mediated Tat activation might lead to an increase in virus production. We compared the amount of viral antigen p24^gag^ in culture medium of HeLa cells transfected with infectious molecular clone pNL4-3 (CXCR4-tropic) with that from cells transfected with pAD8 (CCR5-tropic) combined with three NUCKS1 siRNAs. CXCR4 (pNL4-3)- and CCR5 (pAD8)-tropic virus production was reduced markedly by knockdown of NUCKS1 at different time points (0–5 days) (Figure 5A and B). We next compared the p24 levels from replication competent A3.01 and Jurkat cells infected with pNL4-3 combined with NUCKS1 knockdown. As shown in Figure 5C, the levels of p24 from those cells were decreased by NUCKS1 knockdown. These results indicate that NUCKS1 is required for HIV-1 replication and production involving Tat activity regardless of the tropism.

### NUCKS1 plays an important role in reactivation of provirus of HIV-1 latently infected cells

**Figure 6:**
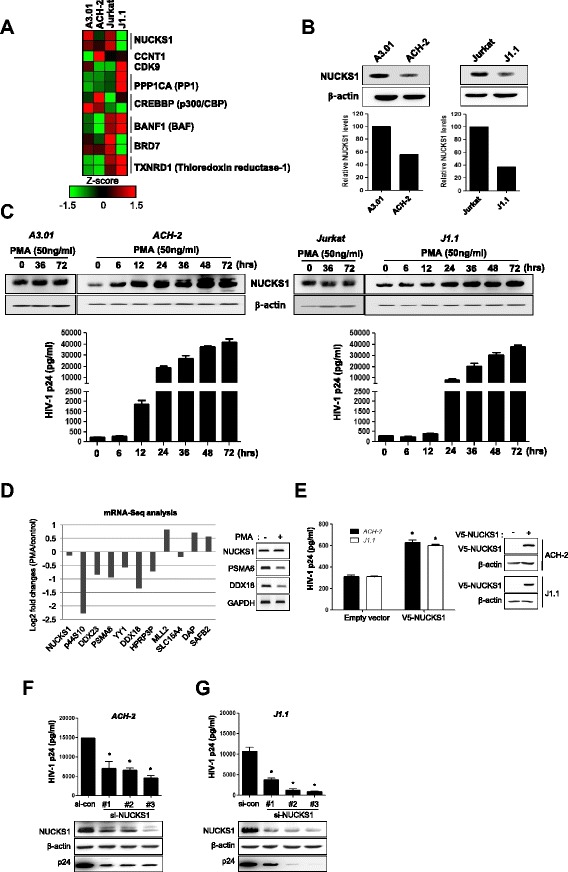
**Requirement of NUCKS1 for reactivation of provirus from HIV-1 latently infected cells. (A)** Heatmap of mRNA expression of NUCKS1 and other Tat-interacting factors. **(B)** Expression level of endogenous NUCKS1 in HIV-1 latent cells (ACH-2 and J1.1) and their parent cell lines (A3.01 and Jurkat) were assessed with Western blotting using anti-NUCKS1 and anti-β-actin antibodies. The protein level of NUCKS1 was calculated by densitometry (Image Gauge Version 4.0, FujiFilm) **(C)** Normal and latently infected cells were treated with PMA (50 ng/ml) for indicated time. PMA-treated cell lysates were assessed by Western blotting using indicated antibodies. The levels of HIV-1 p24 were measured using an HIV-1 p24 ELISA kit. The data are expressed as mean ± SD (n = 3). **(D)** mRNA-sequencing analysis. The RPKM (Reads Per Kilobase of exon model per Million aligned tags) was calculated for each transcript. The Y-axis represents the log2 fold changes between the control and PMA-treated ACH-2 cells. The right panel shows the expression level of NUCKS1 mRNA by RT-PCR. **(E)** ACH-2 and J1.1 cells were electrophorated with V5 or V5-NUCKS1. Three days later, the amount of virion in the supernatant was measured by HIV-1 p24 ELISA kit. The right panel shows the level of NUCKS1 expression by Western blotting. The data are expressed as mean ± SD (n = 3). *, *P* < 0.01 as compared to cells transfected with empty vector. **(F-G)** ACH-2 **(F)** and J1.1 cells **(G)** were electroporated with control or three individual NUCKS1 siRNAs. At 48 h after knockdown, the cells were treated with PMA (50 ng/ml) for 24 h and the levels of HIV-1 p24 were measured using an HIV-1 p24 ELISA kit. The data are expressed as mean ± SD (n = 3). *, *P* < 0.01 as compared to the cells transfected with control siRNA.

HIV-1 latency is characterized by long-term silent infection, which contributes to the HIV-1 pathogenesis [37]. However, the mechanism is not understood fully. Therefore, we attempted to link the NUCKS1-induced HIV-1 replication to viral reactivation from latent HIV-1 infection. We compared the mRNA and protein levels of NUCKS1 between normal and latently HIV-1 infected cell lines. As shown in Figure 6A, the NUCKS1 mRNA level was significantly lower in HIV-1 latently infected cells (ACH-2 and J1.1) compared with the normal parent cells (A3.01 and Jurkat) (Figure 6A). Western blotting of NUCKS1 indicated that latently infected cells expressed less NUCKS1 protein compared with normal cells (Figure 6B).

Next, we investigated whether NUCKS1 expression was increased in latently infected cells during PMA-induced reactivation. The NUCKS1 protein level was increased markedly in latently infected cells in a time-dependent manner during HIV-1 proviral reactivation, as indicated by the HIV p24 antigen production in the supernatant treated with PMA. However, no significant change in NUCKS1 protein level was found in the parent A3.01 and Jurkat cells (Figure 6C). In order to analyze whether the expression level of NUCKS1 mRNA increases due to PMA treatment, mRNA sequencing was performed in a latently infected cell line in the presence or absence of PMA. The transcriptomic analysis showed that the level of NUCKS1 mRNA was not changed under PMA treatment for 48 h, even if other genes were relatively changed as previously reported [38], except SLC15A4 (Figure 6D, Additional file 2: Figure S2 and Additional file 3: Table S1). Additionally, bisulfite sequencing of the CpG island in the NUCKS1 promoter showed that the expression of NUCKS1 was not regulated by epigenetic control (Additional file 4: Figure S3). These data indicated that the transcription of NUCKS1 is not associated with the activation of latently infected cells by PMA treatment. Next, we examined whether NUCKS1 alone induces HIV-1 production without NF-κB activation. As shown in Figure 6E, NUCKS1 overexpression moderately induced HIV-1 production without PMA stimulation. The data may suggest that NUCKS1 can enhance Tat-mediated HIV production without NF-κB activation. To examine further whether NUCKS1 plays an essential role in HIV-1 replication during proviral reactivation in latently infected cells, we conducted NUCKS1 knockdown by electroporation using specific siRNA in latently infected ACH-2 cells and J1.1 cells under PMA-induced reactivation. Western blot analysis using NUCKS1-specific antibody showed the efficiency of the knockdown (Figure 6F and G lower panel). Notably, p24 levels were decreased significantly by knockdown of NUCKS1 in ACH-2 and J1.1 cells during reactivation (Figure 6F and G). The knockdown caused an average decrease of 60% in p24 concentration in ACH-2 cells and 82% in J1.1 cells (Figure 6F and G upper panel). These data show that NUCKS1 is a key regulator of HIV-1 latency and that it plays an important role in HIV-1 reactivation from latently infected cells.

## Discussion

Defining the protein–protein interactions underlying the dynamics of virus–host interactions is critical for understanding the molecular pathogenesis of HIV-1 infection. In this study, NUCKS1 was identified as a novel Tat-interacting protein in a yeast two-hybrid screening system. The interaction between Tat and NUCKS1 was confirmed by coimmunoprecipitation associated with both ectopic and endogenous expression (Figure 1). We have shown here that ectopic expression of NUCKS1 efficiently increased Tat-dependent transactivation of the HIV LTR in a dose-dependent manner. Additionally, siRNA-induced downregulation of endogenous NUCKS1 significantly reduced Tat-mediated transcriptional activation on the HIV-1 LTR promoter (Figure 2). These data indicate that NUCKS1 is a potential candidate as a Tat coactivator that facilitates interactions between Tat and the transcription machinery on the HIV-1 LTR.

Several studies have suggested that NUCKS1 may be involved in facilitating and regulating cellular transcriptional activity. Schaner et al. reported that the ubiquity and abundance of NUCKS1, particularly in rapidly growing and cancer cells, might be associated with its transcriptional role in cell growth [34–36].

NUCKS has been reported to have 19 phosphorylation sites, at least nine of which are phosphorylated by CK2 [39]. Candidates for CK2 substrates are likely to comprise a functional link to transactivation of viral transcription. For example, the Tat-SF1 and TRP-185 cofactors that regulate HIV-1 transcription contain more than 12 and 20 CK2 phosphorylation sites, respectively [17,40]. Similarly, SRB, a protein that stimulates TRP-185 binding to TAR RNA, has 10 putative CK2 sites [41]. CK2 may be linked functionally to some proteins involved in the replication of HIV-1 and stimulation of virally infected cells [42–45]. However, CK2 activity that is specifically linked to NUCKS1 in HIV-1 replication remains a subject for further study.

Although the essential role of Tat during HIV transcriptional activation is well established, NF-κB also stimulates the transcription of HIV-1 LTR in the initiation step. Thus, we could not exclude a possible role of NF-κB activation in NUCKS1-induced HIV-1 transcription together with Tat expression (Figure 2). Therefore, we investigated whether NUCKS1 increases HIV-1 transcriptional activity in association with NF-κB activation. The expression of NUCKS1 had no effect on nuclear translocation of p65, degradation of IκBα, or NF-κB luciferase activity. Furthermore, the expression of NUCKS1 in latently HIV-1 infected cell lines considerably induced HIV-1 production without PMA-induced NF-κB activation (Figures 3A, B and 6E). These data suggested that NUCKS1 increased Tat-mediated transcriptional activity of the HIV-1 LTR in an NF-κB-independent manner. On the other hand, the expression level of NUCKS1 was not enhanced in primary CD4+ T cell activated with anti-CD3/CD28, even though NF-κB was significantly activated in the cells (Additional file 5: Figure S4). These data revealed that NUCKS1 expression was not associated with the NF-κB pathway.

Tat-dependent transactivation of the HIV-1 LTR requires three steps: 1) facilitation of nuclear localization of Tat, 2) Tat–Cyc T1 interaction, and 3) docking of Tat–TAR RNA on the HIV LTR. However, subcellular fractionation and coimmunoprecipitation analysis revealed that NUCKS1 had no effect on Tat nuclear localization and the stability of the Tat–Cyc T1 complex, respectively (Figure 3C and D). This observation suggests that the nuclear localization of Tat and the formation of the Tat–P-TEFb complex are not the functional targets of NUCKS1. Importantly, the ChIP assay showed that knockdown of NUCKS1 decreased the accumulation of Tat at the TAR region on the LTR, suggesting that NUCKS1 promotes Tat-mediated transactivation by increasing Tat–TAR interactions (Figure 4). However, the detailed mechanism by which NUCKS1 regulates Tat–TAR interaction remains unknown. It is possible that NUCKS1 facilitates the interaction of Tat and TAR RNA through chromatin structural remodeling in the LTR region because NUCKS1 is a nuclear protein harboring a DNA-binding domain and it may be involved in multiple transcriptional activities [46].

In this study, we used an HIV-1 production assay to examine the influence of NUCKS1 on HIV-1 replication. Knockdown of NUCKS1 significantly decreased the production of both CXCR4 (pNL4-3)- and CCR5 (pAD8)-tropic viruses (Figure 5A and B). Moreover, the HIV-1 replication assay in A3.01 and the Jurkat cells infected with pNL4-3 showed a significant reduction in the level of HIV-1 p24 caused by NUCKS1 knockdown (Figure 5C). This result showed that NUCKS1 regulated the HIV-1 gene expression directly followed by HIV-1 virion production. Although this was consistent with the NUCKS1-mediated Tat regulation described above, it was insufficient to explain the transcriptional activation leading to proviral reactivation from latently infected cells.

After infection, the provirus successfully integrates into the host chromosome, and HIV-1 latency is established by limiting the levels of several host factors and interfering transcription. We used an mRNA microarray assay to examine HIV-1-latent cell lines (Figure 6). Positively Tat-regulating factors such as CCNT1, protein phospatase 1(PP1), and p300/CBP did not have lower expression levels in latent cells compared with normal cells, suggesting that the basal expression of these factors may not be associated with latent infection [47,48]. Negatively Tat-regulating factors including BanF1, BRD7, and thioredoxin reductase-1 (TXNRD1) did not show consistent differences in their expression patterns between latent and normal cells [49–51]. Of these factors, only NUCKS1, as a positively Tat-regulating factor, was expressed at a lower level in latently infected cells than in normal parent cells (Figure 6A and B). It has been reported that HIV-1 Tat regulating factors such as Cyclin T1 and CDK9 were regulated by T cell receptor activation, which were expressed at an extremely lower level and exhibited restricted activity in primary resting cells [52,53]. Although the expression profile of these factors from our latency model using quickly dividing cell lines was quite different from that of tightly regulated primary resting memory T cells, our data may suggest that a low expression level of NUCKS1 seems to be important for maintaining latent infection characterized by restricted HIV-1 gene expression in the cell line model.

HIV-1 latency is a major barrier to HIV-1 eradication and a critical source for rebound of viral load after the interruption of highly active antiretroviral therapy (HAART). Thus, it is important to identify the host factors that can modulate latent HIV-1 infection [37]. We identified a role of NUCKS1 in the reactivation process in HIV-1 latently infected cells treated with PMA. The protein levels of NUCKS1 were increased markedly in HIV-1 latently infected cells at the same time that HIV-1 replication increased in a time-dependent manner (Figure 6C). The reactivation was increased by overexpression of NUCKS1 without NF-κB activation (Figure 6E) and was reduced markedly by knockdown of NUCKS1 (Figure 6F and G), indicating that NUCKS1 is needed for HIV-1 reactivation. These results show that NUCKS1 might be a novel host factor that increases viral replication by activating ‘silent’ viruses.

## Conclusions

In summary, our study shows that NUCKS1: i) increases the Tat-mediated transcription of the HIV-1 LTR in an NF-κB-independent manner ii) facilitates the interaction of Tat with TAR RNA in the LTR, and iii) plays an important role in the reactivation of latent HIV-1 infection.

Taken together, our data suggest that NUCKS1 plays an important regulatory role in Tat-mediated HIV-1 transcription by facilitating the process of HIV-1 replication and/or awaking from the HIV-1 latency state. Further understanding of the mechanism responsible for NUCKS1-mediated Tat regulation may be helpful in the development of potent new anti-HIV-1 drugs and for strategies for purging HIV-1 from the latency reservoir.

## Methods

### Cells, viruses, and plasmids

HeLa, HEK293, TZM-bl, A3.01, ACH-2, Jurkat, and J1.1 cells were obtained through the NIH AIDS Research and Reference Reagent Program and American Type Culture Collection (ATCC). HeLa, HEK293, and TZM-bl cells were maintained in Dulbecco’s modified Eagle medium (DMEM; Gibco-BRL). ACH-2 and J1.1 are chronically infected cell lines harboring the HIV-1 LAV strain, and A3.01 and Jurkat are the corresponding uninfected parent cell lines. Cells were maintained in RPMI medium (Gibco-BRL). All cells were supplemented with 10% fetal bovine serum (FBS; Gibco-BRL), 1% l-glutamine, and 1% penicillin–streptomycin. Human PBMCs were isolated from blood samples by Ficoll-Hypaque 1.077 (Sigma), and CD4+ T cells were purified from the PBMCs using a CD4+ T cell isolation kit (Miltenyi Biotec) according to the manufacturer’s instructions. The HIV-1 molecular clone pNL4-3 was obtained through the NIH AIDS Research and Reference Reagent Program, and pAD8 was kindly provided by Dr Young-bong Kim, Konkuk University, Seoul, South Korea. Tat-expressing plasmid was constructed by cloning wild-type Tat (HXB2 strain) cDNA into the pcDNA3-Flag (Invitrogen) plasmid. The NUCKS1-expressing plasmid was constructed by cloning the pcDNA3-V5 (Invitrogen) plasmid. The pGL3–LTR plasmid contains a luciferase gene driven by the HIV-1 LTR and was used in the Tat-mediated transactivation reporter assay.

### Knockdown of NUCKS1

Knockdown of NUCKS1 was performed with small interfering RNA (siRNA) corresponding to NUCKS1 (si–NUCKS1) synthesized using the following three oligonucleotide sequences: #1; 5’-GAAGGUUGUUGAUUACUCACAGUUU-3’, #2; 5’-GGAUGAUUCUCACUCAGCAGAGGAU-3’, and #3; 5’-GGCGGCAUCUAAAGCAGCUUCUAAA-3’, and nontargeting siRNA (si–con) purchased from Invitrogen. In brief, HeLa cells were transfected with 200 pmole siRNA using Lipofectamine 2000 reagent (Invitrogen). Twenty-four hours after transfection, cells were cotransfected with pcDNA3–Flag–Tat, pGL3–LTR, and pCMV–LacZ using Lipofectamine 2000 reagent. Forty hours after cotransfection, total cell lysates were harvested for determination of luciferase activity using the luciferase reporter assay system (Promega) on a SpectraMax M5 microplate luminometer (Molecular Devices). Knockdown of HIV-1 latently infected cells (ACH-2 and J1.1 cells) was performed by electroporation using a Nucleofector™ kit X (Lonza) according to the manufacturer’s protocol (18, 40). The expression level of NUCKS1 was analyzed by Western blotting using anti-NUCKS1 antibody (Novus Biologicals).

### Yeast two-hybrid screening

Yeast two-hybrid screening was conducted using the Matchmaker GAL4 two-hybrid system 3 (Clontech) as described previously [54]. In brief, the region containing full-length Tat was generated by PCR, cloned downstream of the GAL4 DNA-binding domain in pGBKT7 (pGBKT7–Tat/bait), and introduced into the yeast strain PBN204 containing three reporters (URA3, ADE2, and LacZ), which are under the control of different GAL promoters. The transformants of the Tat bait and the human thymus cDNA activation domain library were screened on selection medium lacking leucine, tryptophan, and uracil, which support the growth of yeast when the bait plasmid and the prey proteins interact with each other. After selection of the putative Tat-binding protein, NUCKS1, the amplified NUCKS1-expressing prey plasmid was reintroduced into the yeast PBN204 strain with the Tat bait plasmid to confirm the interaction of Tat–NUCKS1.

### Luciferase reporter assays

HeLa cells were cultured on 12-well plates (2.5 × 10^5^ cells/well) for 1 day before transfection. The cells were transfected with 0.05 μg of pcDNA3–Flag–Tat, 0.2 μg of pGL3–LTR–Luc, and different amounts of pcDNA3–V5–NUCKS1 plasmids using Lipofectamine 2000 reagent (Invitrogen). pCMV–LacZ (0.02 μg) was cotransfected as a transfection control. The luciferase assay was performed as described previously [55]. In brief, the cell medium was replaced with fresh medium 4 h after transfection. Twenty-four hours after transfection, luciferase activity was measured using a luciferase reporter assay system (Promega) in a DTX 880 MultiMode Detector (Beckman Coulter). Luciferase activity was normalized to β-galactosidase activity. The data are represented as mean ± SD (n = 3).

### Coimmunoprecipitation

The coimmunoprecipitation assay was performed as described previously [56]. Briefly, HEK293 cells were transfected with Tat-expressing plasmids (pcDNA3–Flag–Tat) and NUCKS1-expressing plasmids (pcDNA3–V5–NUCKS1) using Lipofectamine 2000 (Invitrogen). After 48 h, cells were lysed in immunoprecipitation buffer (250 mM NaCl, 0.5% NP-40, 50 mM Tris-HCl [pH 7.5], 5 mM EDTA, 1 mM Na_3_VO_4_, 10 mM NaF, and protease inhibitor cocktail). Cell lysates were incubated with anti-Flag monoclonal antibody (M2; Sigma) at 4°C. After overnight incubation, protein A/G agarose beads (Santa Cruz Biotechnology) were added, and the mixture was incubated for 1 h at 4°C and then centrifuged at 2,500 × *g* at 4°C for 2 min. The supernatant was removed, and the beads were washed carefully four times with immunoprecipitation buffer. Finally, the beads were resuspended in 5 × SDS sample buffer (125 mM Tris-HCl [pH 6.8], 4% SDS, 20% glycerol, and 2% β-mercaptoethanol) and analyzed by Western blotting.

### Western blotting

Total cell lysates were prepared using lysis buffer containing 50 mM (pH 7.4) Tris-HCl, 1% Nonidet P-40, 150 mM NaCl, 2.5% deoxycholate, 1 mM phenylmethylsulfonyl fluoride, and protease inhibitor (Roche). Cell lysates were mixed with 5 × SDS sample buffer and then loaded on a gel for SDS-PAGE. Proteins were subsequently transferred to a polyvinylidene difluoride membrane (Millipore). The membrane was blocked with 5% nonfat milk and incubated with primary antibodies at 4°C overnight. The primary antibodies used were anti-Flag M2 (Sigma), anti-V5 (Invitrogen), anti-NUCKS1 (Novus Biologicals), anti-p65 subunit of NF-κB (Cell Signaling Technology), anti-IκB-α (Cell Signaling Technology), anti-CycT1 (Santa Cruz Biotechnology), anti-β-actin (Sigma), anti-α-tubulin (Sigma), and anti-lamin B (Santa Cruz Biotechnology). The secondary antibodies used were rabbit anti-mouse antibody and goat anti-rabbit antibody (both from Sigma) conjugated with horseradish peroxidase (HRP). ECL Western blotting detection reagents (Millipore) were used for signal detection with HRP-conjugated anti-mouse and anti-rabbit IgG. Chemiluminescence was visualized using Image Lab™ software (Bio-Rad) according to the manufacturer’s protocols. The relative intensities of the NUCKS1 proteins were measured using the Fujifilm Image Gauge V 4.0 programme (Fujifilm).

### Chromatin immunoprecipitation (ChIP) assay and quantitative real-time PCR

The ChIP assay was performed in HeLa and TZM-bl cells using a ChIP assay kit (Cell Signaling Technology) according to the manufacturer’s protocol. Briefly, cells were transfected with Tat-expressing plasmid (pcDNA3–Flag–Tat) after knockdown of NUCKS1. Chromatin was then obtained from the formaldehyde-fixed cells, and the chromatin was immunoprecipitated with anti-Flag antibody or control mouse IgG antibody. The eluted material was reverse cross-linked, and DNA was precipitated with ethanol and isolated by spin column purification. The purified DNA was then analyzed by real-time PCR using primer sets corresponding to the TAR region of the HIV-1 LTR. The primers used were TAR-F, 5’-AGCTTTCTACAAGGGACTTTCCGC-3’ and TAR-R, 5’-ATTGAGGCTTAAGCAGTGGGTTCC-3’. Real-time PCR was performed using a SYBR Green PCR kit (TAKARA) in a 7500 Real-Time PCR System (Applied Biosystems (ABI)) using 7500 software, version 2.0.1. Melting curve analysis was performed to ensure the amplification of a single product. The data were analyzed according to the comparative Ct method, were normalized to the IgG control, and are reported as the fold change in binding relative to control siRNA. Relative quantification (RQ) = 2 ^–ΔΔCt^, where ΔCt = Ct_Target_– Ct_Control_, and ΔΔCt = ΔCt_treated_– Ct_untreated_.

### Gene expression microarray data analysis

Gene expression microarray analysis was performed using the Illumina Human HT-12 v4 Expression BeadChip (Illumina). The Illumina Total Prep RNA Amplification Kit (Ambion) was used to prepare biotinylated cRNAs from 0.55 μg of total RNA. After fragmentation, 0.75 μg of cRNAs were mixed with hybridization buffer and hybridized to the BeadChip. Following incubation in the hybridization oven, the hybridized arrays were washed. The microarray images were scanned using the Illumina Bead array Reader Confocal Scanner. Array data export processing was performed using Illumina GenomeStudio v2009.2 (Gene Expression Module v1.5.4). All datasets were normalized using the quantile normalization method. Average values were used for replicated data sets. The Z-score was calculated by subtracting the population mean from an individual expression value and then dividing the difference by the population standard deviation (SD).

### mRNA-Sequencing

The total RNA was extracted using Tri-solution (BSK), and the mRNA was isolated using an Oligotex mRNA mini kit (Qiagen). The fragmented mRNA was converted into cDNA using a random hexamer and Superscript III reverse transcriptase (Invitrogen). Following our previous study [57], the mRNA sequencing library was constructed and sequenced on the Genome Analyzer IIx (Illumina). Sequencing reads were aligned by the TopHat v2.0.9 alignment program [58] to the human reference sequence (hg18 RefSeq). The RPKM (*Reads Per Kilobase of exon per Million aligned tags*) values for each transcript were calculated by normalizing with the length of the gene transcript and the total number of aligned tags.

### Quantitative RT- PCR

The total RNA was extracted using Tri-solution (BSK), and the mRNA was isolated using an Oligotex mRNA mini kit (Qiagen). The mRNA was converted into cDNA using a random hexamer and Superscript III reverse transcriptase (Invitrogen). PCR was carried out using the following primers; PSMA6-F: 5’-ACTGCAATTACATGCCTGTCT-3’, PSMA6-R: 5’-CACAGGTAAGTGGCATCACG-3’, DDX18-F: 5’-AAGATCGAGAAGCGGAACCT-3’, DDX18-R: 5’-TCCCACTGCTTCTTGAGACA-3’ NUCKS1-F: 5’-TGCCCAAACCCAGACTAAAG-3’, NUCKS1-R: 5’-GACCCTTCATCCCCAGATTT-3’, GAPDH-F: 5’-GCAGCCTCCCGCTTCGCTC-3’, GAPDH-R: 5’-GCCAGCATCGCCCCACTTGA-3’.

### Viral production assays

For HIV-1 production assays in a single round, HeLa cells (2 × 10^5^ cells/well) were transfection with siRNA as described above. After 24 h, the cells were transfected with pNL4-3 (300 ng) or pAD8 (300 ng). One day after transfection, virus-containing supernatants were harvested every day for 5 days and filtered to remove debris. The amount of virions in the supernatants was determined using an HIV-1 p24 enzyme-linked immunosorbent assay (ELISA) kit (PerkinElmer) according to the manufacturer’s protocol with minor modification. Briefly, all samples were diluted in medium, and 200 μl of each diluted sample was used for the assay. The culture medium from untransfected cells was used as the negative control. The results were quantified by plotting absorbance values and comparing these with a standard curve. All assays were performed in triplicate. The data are represented as mean ± SD (n = 3).

### Viral replication assay

1 × 10^6^ 293 T cells cells were transfected with 2 μg of pNL4-3. Three days after the transfection, virus-containing supernatants were harvested. The amount of virions in the supernatants was determined using an HIV-1 p24 enzyme-linked immunosorbent assay (ELISA) kit (PerkinElmer). For HIV-1 infection, 1 × 10^6^ Jurkat and A3.01 cells were re-suspended with 0.5 μg of pNL4-3 and centrifuged at 2,500 rpm for 90 min at 30°C. Five days after infection, the amount of virions in the supernatants was determined by HIV-1 p24 ELISA kit.

### Statistical analysis

The data were analysed for statistical significance and expressed as mean values ± SD. The mean values were compared using Student’s t-test with GraphPad Instat Software. P-values of < 0.05 were considered statistically significant.

## Additional files

Additional file 1: Figure S1. The association between NUCKS1 and Tat binding to TAR RNA. (A–B) The HeLa cells transiently transfected with HIV-1 LTR (A) or TZM-bl cells containing an integrated HIV-1 LTR (B) were transfected with control siRNA or three individual NUCKS1 siRNAs. At 24 h after knockdown, these cells were transiently transfected with the Flag–Tat expression plasmid and cultured for an additional 24 h. The cells were cross-linked with formaldehyde, and chromatin immuno-precipitation was performed using IgG control or anti-Flag antibodies. Quantitative real-time PCR was performed with the primer sets corresponding to the upstream and downstream of the TAR region of the HIV-1 LTR. PCR was carried out using the following primers; TAR-F: 5’-AGCTTTCTACAAGGGACTTTCCGC-3’ and TAR-R, 5’-ATTGAGGCTTAAGCAGTGGGTTCC-3’. Up TAR-F: 5’-CACACAAGGCTACTTCCCTGA-3’, Up TAR-R: 5’-GGCCATGTGATGAAATGCTA-3’, Down TAR-F: 5’-TGTGTGCCCGTCTGTTGTGT-3’, Down TAR-R: 5’ -CCTGCGTCGAGAGAGCTC-3’. ; the data were normalized to the IgG control antibodies and are expressed as the fold change of Tat binding activity compared with the control siRNA-treated samples. The data were expressed as mean ± SD (n=3). Additional file 2: Figure S2. The mRNA expression values for Tat-binding genes in ACH-2 cells by PMA treatment. The RPKM (Reads Per Kilobase of exon model per Million aligned tags) was calculated for each transcript. The expression value for the ACH-2 control is shown in white, the ACH-2 PMA in black. Additional file 3: Table S1. Functional annotations for the significant DEGs in PMA-treated ACH-2 cells. Additional file 4: Figure S3. Bisulfite sequencing of CpG island in NUCKS1 the promoter region of ACH-2 cells. The PCR region includes 13 individual CpG sites from -1160 bp to -983 bp relative to the transcription start site of the NUCKS1 gene. The open circles represent un-methylated CpG, while the filled circle represents methylated CpG. UT; PMA-untreated. Additional file 5: Figure S4. The level of NUCKS1 in resting and activated primary CD4+ T cells. The resting CD4+ T cells isolated from PBMCs were activated with anti-CD3/CD28 antibodies and IL-2 for 1 h. Nucleus and cytoplasm were fractionated and protein level was assessed by Western blotting using anti-p65, -Iκb, and -NUCKS1 antibodies. The nuclear and cytoplasmic fractions were evaluated using anti-lamin B and -tubulin antibodies, respectively.
