# Supplementary material for: NUCKS1, a novel Tat coactivator, plays a crucial role in HIV-1 replication by increasing Tat-mediated viral transcription on the HIV-1 LTR promoter
Source: Retrovirology. 2014 Aug 13;11:67. doi: 10.1186/s12977-014-0067-y (PMC4181878; doi:10.1186/s12977-014-0067-y)
Supplement: Additional file 1: Figure S1. — The association between NUCKS1 and Tat binding to TAR RNA. (A–B) The HeLa cells transiently transfected with HIV-1 LTR (A) or TZM-bl cells containing an integrated HIV-1 LTR (B) were transfected with control siRNA or three individual NUCKS1 siRNAs. At 24 h after knockdown, these cells were transiently transfected with the Flag–Tat expression plasmid and cultured for an additional 24 h. The cells were cross-linked with formaldehyde, and chromatin immuno-precipitation was performed using IgG control or anti-Flag antibodies. Quantitative real-time PCR was performed with the primer sets corresponding to the upstream and downstream of the TAR region of the HIV-1 LTR. PCR was carried out using the following primers; TAR-F: 5’-AGCTTTCTACAAGGGACTTTCCGC-3’ and TAR-R, 5’-ATTGAGGCTTAAGCAGTGGGTTCC-3’. Up TAR-F: 5’-CACACAAGGCTACTTCCCTGA-3’, Up TAR-R: 5’-GGCCATGTGATGAAATGCTA-3’, Down TAR-F: 5’-TGTGTGCCCGTCTGTTGTGT-3’, Down TAR-R: 5’ -CCTGCGTCGAGAGAGCTC-3’. ; the data were normalized to the IgG control antibodies and are expressed as the fold change of Tat binding activity compared with the control siRNA-treated samples. The data were expressed as mean ± SD (n=3). [file 12977_2014_67_MOESM1_ESM.pdf]

Additional file 1: Figure S1

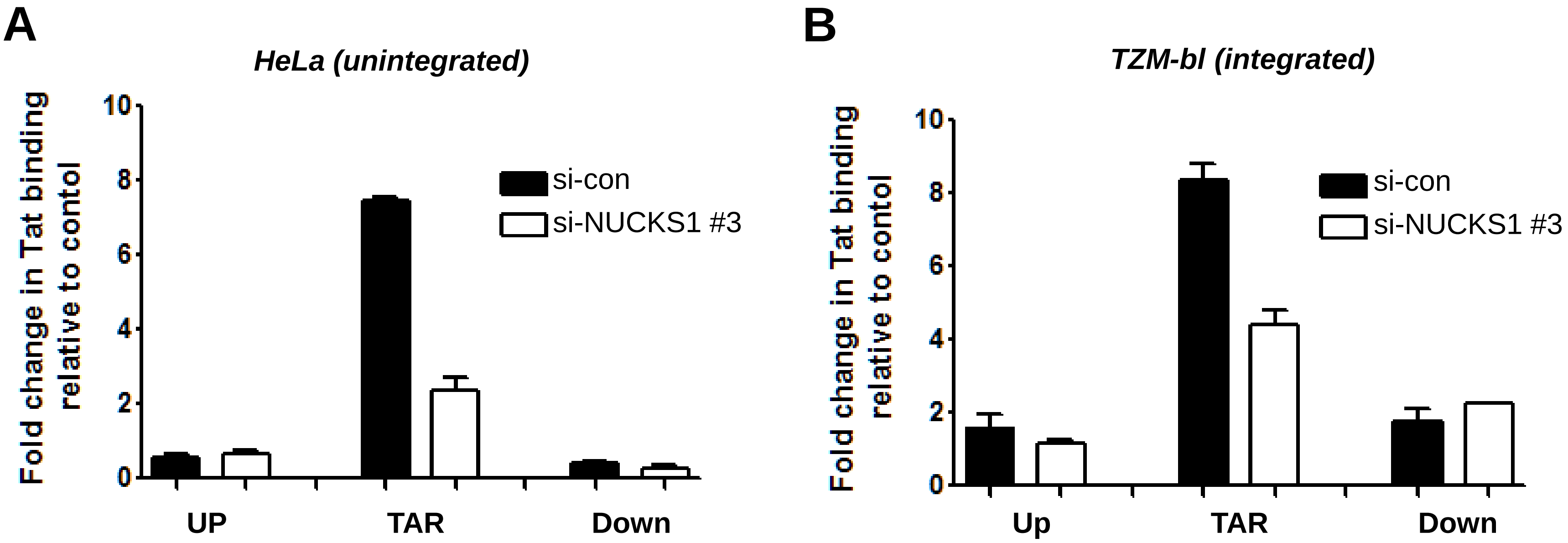

Additional file 1: Figure S1. The association between NUCKS1 and Tat binding to TAR RNA. (A–B) The HeLa cells transiently transfected with HIV-1 LTR (A) or TZM-bl cells containing an integrated HIV-1 LTR (B) were transfected with control siRNA or three individual NUCKS1 siRNAs. At 24h after knockdown, these cells were transiently transfected with the Flag–Tat expression plasmid and cultured for an additional 24h. The cells were cross-linked with formaldehyde, and chromatin immuno-precipitation was performed using IgG control or anti-Flag antibodies. Quantitative real-time PCR was performed with the primer sets corresponding to the upstream and downstream of the TAR region of the HIV-1 LTR. PCR was carried out using the following primers; TAR-F: 5′-AGCTTTCTACAAGGGACTTTCCGC-3′ and TAR-R, 5′-ATTGAGGCTTAAGCAGTGGGTTCC-3′. Up TAR-F: 5′-CACACAAGGCTACTTCCCTGA-3′, Up TAR-R: 5′-GGCCATGTGATGAAATGCTA-3′, Down TAR-F: 5'-TGTGTGCCCGTCTGTTGTGT-3′, Down TAR-R: 5′ -CCTGCGTCGAGAGAGCTC-3′. ; the data were normalized to the IgG control antibodies and are expressed as the fold change of Tat binding activity compared with the control siRNA-treated samples. The data were expressed as mean ± SD (n=3).
