# Supplementary material for: NUCKS1, a novel Tat coactivator, plays a crucial role in HIV-1 replication by increasing Tat-mediated viral transcription on the HIV-1 LTR promoter
Source: Retrovirology. 2014 Aug 13;11:67. doi: 10.1186/s12977-014-0067-y (PMC4181878; doi:10.1186/s12977-014-0067-y)
Supplement: Additional file 2: Figure S2. — The mRNA expression values for Tat-binding genes in ACH-2 cells by PMA treatment. The RPKM (Reads Per Kilobase of exon model per Million aligned tags) was calculated for each transcript. The expression value for the ACH-2 control is shown in white, the ACH-2 PMA in black. [file 12977_2014_67_MOESM2_ESM.pdf]

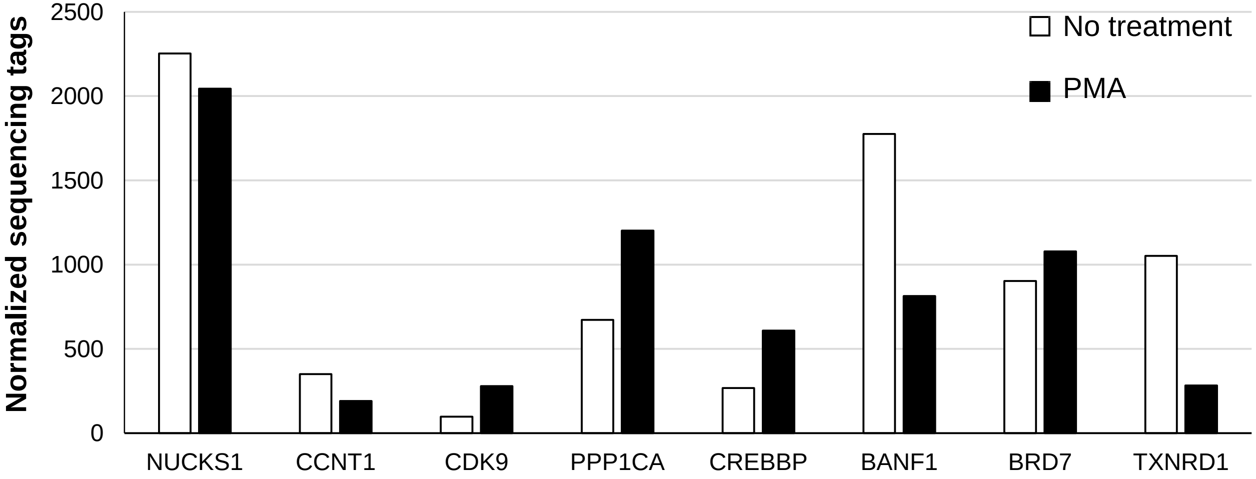

Additional file 2: Figure S2. The mRNA expression values for Tat-binding genes in ACH-2 cells by PMA treatment. The RPKM (Reads Per Kilobase of exon model per Million aligned tags) was calculated for each transcript. The expression value for the ACH-2 control is shown in white, the ACH-2 PMA in black.
