# Supplementary material for: NUCKS1, a novel Tat coactivator, plays a crucial role in HIV-1 replication by increasing Tat-mediated viral transcription on the HIV-1 LTR promoter
Source: Retrovirology. 2014 Aug 13;11:67. doi: 10.1186/s12977-014-0067-y (PMC4181878; doi:10.1186/s12977-014-0067-y)
Supplement: Additional file 3: Table S1. — Functional annotations for the significant DEGs in PMA-treated ACH-2 cells. [file 12977_2014_67_MOESM3_ESM.pdf]

Additional file 3: Table S1. Functional annotations for the significant DEGs in PMA-treated ACH-2 cells

Up by PMA (1737 DEGs)

GO term	Count	%	P-Value	Benjamini
regulation of small GTPase mediated signal transduction	36	3.2	1.30E-06	4.00E-03
regulation of Ras protein signal transduction	32	2.8	1.50E-06	2.20E-03
immune response	71	6.2	3.00E-06	3.00E-03
inflammatory response	39	3.4	2.90E-05	2.20E-02
myeloid leukocyte activation	12	1.1	4.80E-05	2.90E-02
regulation of Rho protein signal transduction	18	1.6	5.00E-05	2.50E-02
response to wounding	54	4.7	7.10E-05	3.00E-02
negative regulation of endocytosis	7	0.6	1.20E-04	4.40E-02
actin cytoskeleton organization	29	2.5	1.20E-04	3.90E-02
leukocyte activation	30	2.6	1.60E-04	4.90E-02

Down by PMA (798 DEGs)

GO term	Count	%	P-Value	Benjamini
ncRNA metabolic process	22	3.8	2.20E-06	4.10E-03
ncRNA processing	19	3.3	5.60E-06	5.20E-03
regulation of protein ubiquitination	13	2.3	2.40E-05	1.40E-02
RNA processing	34	5.9	3.20E-05	1.40E-02
ribonucleoprotein complex biogenesis	17	3	5.00E-05	1.80E-02
ribosome biogenesis	13	2.3	1.70E-04	5.00E-02
tRNA processing	10	1.7	2.70E-04	6.90E-02
cell cycle process	32	5.6	3.10E-04	6.80E-02
rRNA metabolic process	11	1.9	3.60E-04	7.20E-02
regulation of ligase activity	10	1.7	4.40E-04	7.80E-02

Note: The DEGs were identified when the genes showed a more than fourfold change and the tag difference was more than thirty. Using the DAVID database, GO terms significantly enriched with DEGs were identified, and the top ten GO terms are shown.
