# Supplementary material for: NUCKS1, a novel Tat coactivator, plays a crucial role in HIV-1 replication by increasing Tat-mediated viral transcription on the HIV-1 LTR promoter
Source: Retrovirology. 2014 Aug 13;11:67. doi: 10.1186/s12977-014-0067-y (PMC4181878; doi:10.1186/s12977-014-0067-y)
Supplement: Additional file 4: Figure S3. — Bisulfite sequencing of CpG island in NUCKS1 the promoter region of ACH-2 cells. The PCR region includes 13 individual CpG sites from -1160 bp to -983 bp relative to the transcription start site of the NUCKS1 gene. The open circles represent un-methylated CpG, while the filled circle represents methylated CpG. UT; PMA-untreated. [file 12977_2014_67_MOESM4_ESM.pdf]

# Additional file 4: Figure S3

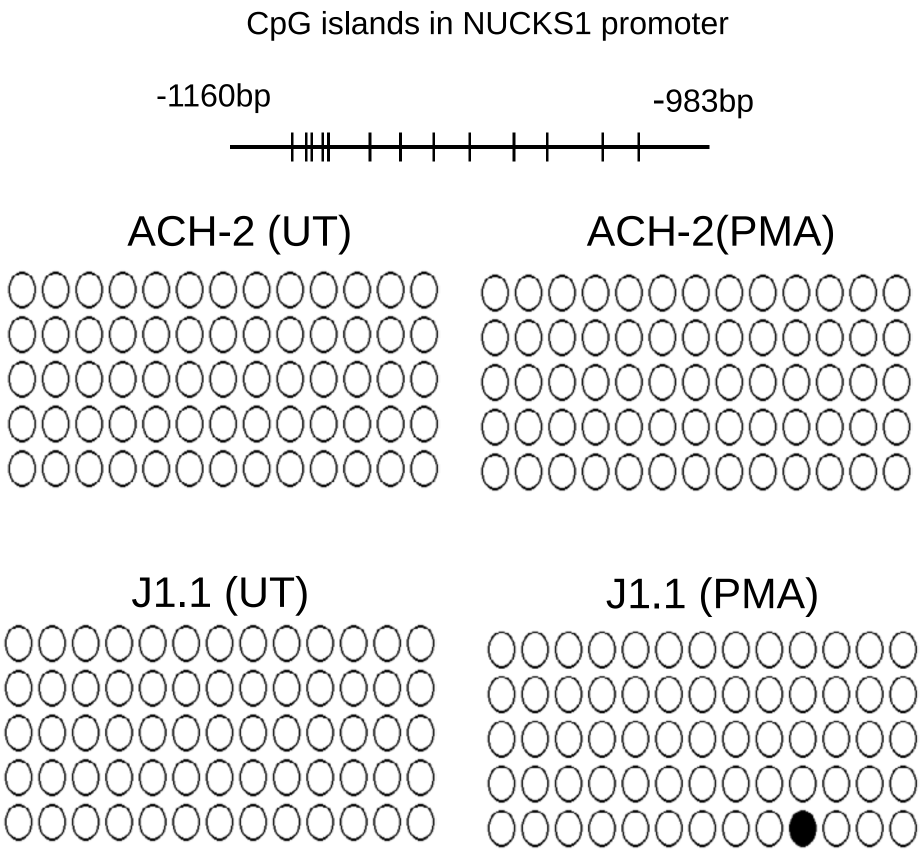


**Additional file 4: Figure S3.** Bisulfite sequencing of CpG island in NUCKS1 the promoter region of ACH-2 cells. The PCR region includes 13 individual CpG sites from -1160bp to -983bp relative to the transcription start site of the NUCKS1 gene. The open circles represent un-methylated CpG, while the filled circle represents methylated CpG. UT; PMA-untreated
