# Supplementary material for: NUCKS1, a novel Tat coactivator, plays a crucial role in HIV-1 replication by increasing Tat-mediated viral transcription on the HIV-1 LTR promoter
Source: Retrovirology. 2014 Aug 13;11:67. doi: 10.1186/s12977-014-0067-y (PMC4181878; doi:10.1186/s12977-014-0067-y)
Supplement: Additional file 5: Figure S4. — The level of NUCKS1 in resting and activated primary CD4+ T cells. The resting CD4+ T cells isolated from PBMCs were activated with anti-CD3/CD28 antibodies and IL-2 for 1 h. Nucleus and cytoplasm were fractionated and protein level was assessed by Western blotting using anti-p65, -Iκb, and -NUCKS1 antibodies. The nuclear and cytoplasmic fractions were evaluated using anti-lamin B and -tubulin antibodies, respectively. [file 12977_2014_67_MOESM5_ESM.pdf]

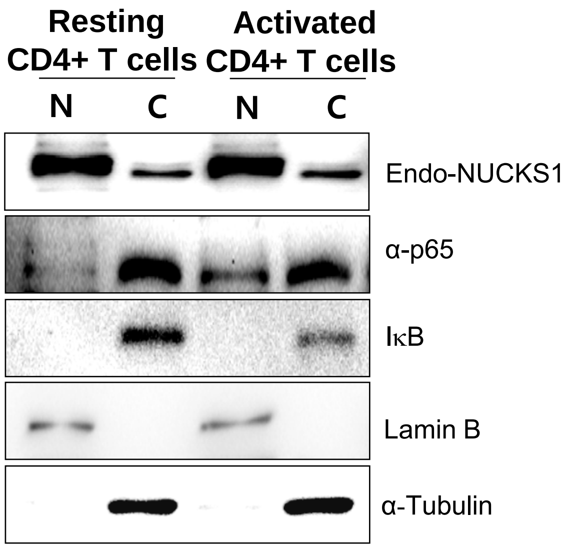

Additional file 5: Figure S4. The level of NUCKS1 in resting and activated primary CD4+ T cells. The resting CD4+ T cells isolated from PBMCs were activated with anti-CD3/CD28 antibodies and IL-2 for 1h. Nucleus and cytoplasm were fractionated and protein level was assessed by Western blotting using anti-p65, -Iκb, and -NUCKS1 antibodies. The nuclear and cytoplasmic fractions were evaluated using anti-lamin B and -tubulin antibodies, respectively.
